# Technic of Wiring Corresponding Teeth of Superior and Inferior Maxilla in Fracture of Inferior Maxilla

**Published:** 1919

**Authors:** 


### FACIO-MAXILLARY SURGERY

---

**Technic of Wiring Corresponding Teeth of Superior and Inferior Maxilla in Fracture of Inferior Maxilla.** By E. BUTLER. *The American Journal of Surgery*, January, 1918.

The mouth is cleansed by irrigating with a weak peroxide of hydrogen solution followed by sterile water. Clots of food may be loosened with a wooden applicator mounted with cotton. If the patient is excitable, anesthesia (gas and oxygen or ether) is required. The fracture is then reduced, and any loose tooth in the line of fracture removed. When the occlusion is correct, the teeth to be wired are selected and the jaws separated again.

Six-centimeter lengths of copper wire, No. 26 or smaller, are cut and the teeth of the superior maxilla prepared first. The first upper molar is taken, for example. The cheek is gently retracted with the finger or Parker retractor, and the wire slipped through the proximal interdental space. Each end is grasped with a clamp and the protruding ends twisted, the first twist fixing the wire around the body of the tooth and again until six twists are made. If the two ends are grasped in one clamp and twisted, thc tension from torsion snaps the wire before it clinches the tooth. The wire must be proximal to the shoulder of the tooth and take the shape of the circumference. The remaining selected teeth are prepared in the same manner.

Once the wires are in place, the inferior jaw is forced into position of correct occlusion, the wires of the corresponding teeth are twisted or hooked together, the superfluous portions cut off, and the rough ends turned against the teeth and guarded with dental wax or rubber. If the wires are properly applied, no adjustment from day to day is necessary.

The wires may be removed from the 14th to the 21st day; the longer period is advisable unless their removal is deemed judicious before.

